# Serum neurofilament light chain in hydrocephalus and surgical controls: baseline comparison and 24-hour perioperative dynamics under general anesthesia

**DOI:** 10.1007/s10143-026-04254-5

**Published:** 2026-03-31

**Authors:** Miroslav Cihlo, Pavel Trávníček, Alena Tichá, Radomír Hyšpler, Marta Kalousová, Tomáš Česák, Karel Zadrobílek, Lucie Kukrálová, Pavel Dostál, Tomáš Zima, Michaela Sluková, Vlasta Dostálová

**Affiliations:** 1 Department of Neurosurgery, Faculty of Medicine in Hradec Kralove, Charles University, Hradec Kralove, Czech Republic; 2 Department of Neurosurgery, University Hospital Hradec Kralove, Hradec Kralove, Czech Republic; 3 Department of Biochemistry, University Hospital Hradec Kralove, Hradec Kralove, Czech Republic; 4 Institute of Medical Biochemistry and Laboratory Diagnostics, First Faculty of Medicine, Charles University and General University Hospital in Prague, Prague, Czech Republic; 5 Department of Anaesthesiology and Intensive Care Medicine, Faculty of Medicine in Hradec Kralove, Charles University, Hradec Kralove, Czech Republic; 6 Department of Anaesthesiology and Intensive Care Medicine, University Hospital Hradec Kralove, Hradec Kralove, Czech Republic

**Keywords:** Idiopathic normal-pressure hydrocephalus, Neurofilament light chain, Serum biomarkers, Anesthesia, Perioperative period

## Abstract

Background/Objectives neurofilament light chain (NfL) is a blood-accessible marker of neuroaxonal injury. We assessed the clinical utility of serum NfL in adults with idiopathic normal-pressure hydrocephalus (iNPH). Methods in a single-center prospective cohort (registered at ClinicalTrials.gov; partial overlap with a prior registry detailed in Supplementary Table S1), we enrolled 41 adults aged ≥ 50 years. The hydrocephalus arm included only anticipated ventriculoperitoneal (VP) shunt responders (Group A); non-hydrocephalus surgical patients served as controls (Group B). The primary analysis compared log-transformed serum NfL between groups using Welch’s t-test. A prespecified paired subset was sampled before premedication (baseline) and 24 h after surgery start. As a sensitivity analysis, we fitted age-aware linear models for log(NfL) with group as the exposure and age and sex as covariates. Results serum NfL was right-skewed; analyses used log-transformed values. The primary between-group contrast showed no difference (geometric mean ratio [GMR] 1.03; 95% CI 0.51–2.10; p = 0.93). In age-adjusted models (log[NfL] ~ group + age + sex), the between-group difference remained non-significant with an effect magnitude comparable to the unadjusted GMR. In paired analyses, serum NfL did not change from baseline to 24 h overall (24 h vs. baseline GMR 1.13; 95% CI 0.89–1.45; p = 0.305) and remained non-significant within both Group A (n = 10) and Group B (n = 11). Conclusions in adults ≥ 50 years, serum NfL neither distinguished anticipated VP-shunt responders with iNPH from surgical controls nor exhibited an acute change at 24 h after surgery start under general anesthesia. These findings suggest limited standalone diagnostic/prognostic utility of serum NfL in this setting. Interpretation should be age-aware and integrated with complementary clinical data and biomarkers; future studies should use standardized sampling and multimodal panels to refine risk stratification.

**Supplementary Information:**

The online version contains supplementary material available at 10.1007/s10143-026-04254-5.

## Introduction

Neurofilament light chain (NfL) is the smallest subunit of neuronal neurofilaments that form the axonal cytoskeleton. Axonal disruption—due to neurodegeneration, ischemia, trauma, or inflammation—releases NfL into interstitial fluid and cerebrospinal fluid (CSF), with subsequent spillover into blood. Historically quantified in CSF by ELISA, NfL can now be measured in plasma/serum using ultrasensitive single-molecule assays, enabling blood–CSF correlations in idiopathic normal-pressure hydrocephalus (iNPH) and other disorders [1–8].

As a fluid biomarker, NfL reflects the burden of axonal injury and therefore provides a quantitative, albeit non-specific, index of neuro-axonal damage across diseases [1]. In iNPH, CSF NfL is typically elevated versus non-hydrocephalus surgical controls and serum NfL correlates with CSF levels; nevertheless, NfL often overlaps with levels observed in Alzheimer’s disease and subcortical ischemic vascular disease, limiting stand-alone diagnostic precision and motivating multimarker approaches (e.g., with Aβ and tau species) [2, 9–13]. Longitudinally, NfL tracks disease activity in several conditions and can change with treatment, but effect direction and timing may vary by pathology and sampling window [2, 14, 15].

Perioperative studies using ultrasensitive assays show that serum NfL commonly rises after surgery—particularly in older or high-risk patients—whereas exposure to general anesthesia without surgery does not produce an early increase. Serial sampling demonstrates progressive rises peaking around 24–48 h after procedure start; other markers (e.g., tau, GFAP) may show parallel or more variable trajectories with age-dependent baselines [16–19]. Higher perioperative NfL has been associated with postoperative MRI evidence of covert/overt ischemic brain injury and with delirium severity, although causal pathways and durability remain uncertain; heterogeneity in procedures, timing, and platforms limits direct comparisons and underscores the need for standardized, age-aware interpretation [20–22].

The aim of this study was to evaluate whether serum NfL distinguishes VP-shunt responders with iNPH from non-hydrocephalus surgical controls, and to assess short-term change at 24 h after surgery start under general anesthesia in serum NfL in a predefined paired subset sampled at baseline and 24 h after surgery start.

## Materials and methods

This was a single-center, prospective observational cohort conducted at the University Hospital Hradec Králové, Czech Republic. The study was approved by the institutional Ethics Committee (approval No. 202311 P02, December 10, 2023) and registered at ClinicalTrials.gov (NCT05399602). All participants provided written informed consent prior to any study procedures.

Participants were recruited between January 2024 and June 2025. Potential candidates were identified by a neurologist and evaluated in a specialized outpatient clinic. Ethnicity was not separately recorded; all participants were recruited in the Czech Republic and were Czech nationals.

**Inclusion criteria (common)**.

- Age ≥ 50 years.
- Communicating hydrocephalus on MRI/CT (for the hydrocephalus arm).
- MMSE > 10.
- No structural brain lesion on MRI/CT.

**Exclusion criteria (common)**.

- Non-communicating hydrocephalus.
- Structural brain lesions (tumor, contusion, aneurysm).
- MMSE ≤ 10.
- Life expectancy < 1 year.
- Pre-existing dementia (e.g., Alzheimer’s disease, vascular dementia).

**Group A** (iNPH—anticipated VP-shunt responders): All patients with suspected idiopathic normal-pressure hydrocephalus (iNPH) underwent a standardized stepwise CSF evaluation to determine anticipated responsiveness to ventriculoperitoneal (VP) shunting, including the tap test (TT), lumbar infusion test (LIT), and/or external lumbar drainage (ELD). Gait and balance were assessed using a shortened 5-meter gait test (number of steps, time to 5 m, stride length), together with the Mini-Mental State Examination (MMSE) and continence status. The iNPH Scale was calculated immediately before the initial lumbar puncture and 4 h thereafter; following TT, gait and MMSE were reassessed at 24 h, and after ELD at 3 days. Patients meeting predefined criteria for a positive CSF-test response proceeded to VP shunt surgery. Only patients scheduled for shunting on this basis (i.e., anticipated responders) were eligible for inclusion in Group A; anticipated non-responders were not included in the present analysis.

**Group B** (non-hydrocephalus surgical controls): Adults ≥ 50 years without a history of hydrocephalus or dementia were enrolled if scheduled for single-level anterior cervical discectomy and fusion (ACDF) under general anesthesia with planned duration < 90 min. Exclusion criteria included dural sac/root involvement, operative time ≥ 90 min, or intraoperative CSF leak. Single-level ACDF was selected as a control procedure to provide comparable exposure to general anesthesia while minimizing direct intracranial or intradural CNS manipulation.

### Anesthesia protocol

Anesthesia was conducted identically in both groups. Induction included propofol titrated in 50-mg increments (up to 2 mg/kg), atracurium 0.5 mg/kg, and sufentanil 0.1 µg/kg. Maintenance used desflurane under low fresh-gas flow with end-tidal control (GE Healthcare). Depth of anesthesia was targeted to State Entropy 40–60 (GE Entropy module). Analgesia was supplemented with a sufentanil bolus (same as induction dose) if the Surgical Pleth Index (SPI) increased by ≥ 10 points from the baseline measured 5 min after intubation. Neuromuscular blockade was deepened with atracurium 0.1 mg/kg as needed, with quantitative neuromuscular monitoring (NMT; GE Healthcare). Emergence and extubation followed institutional routine once standard criteria were met.

### Perioperative exclusion criteria

sustained mean arterial pressure < 65 mmHg for > 5 min, intraoperative blood loss ≥ 500 mL, or arrhythmia requiring pharmacologic treatment.

### Serum sampling occurred twice

(i) on the day of surgery before premedication (baseline), and (ii) 24 h after the start of the surgical procedure. Blood was collected into tubes without anticoagulant. After clotting at room temperature for 20 min blood was centrifuged.

at 2000 g for 10 min. Resulting serum was stored at -80 °C until analysis and thawed just once for the analysis of NfL. Serum NfL was quantified using Simoa (Single molecule array, Quanterix, Billerica, MA, USA) according to the manufacturer’s instructions.

### Statistical analysis

The primary comparison contrasted Group A versus Group B at baseline on the log scale using Welch’s t-test (allowing unequal variances). Effect sizes are reported as geometric mean ratios (GMR = exp[Δ mean log(NfL)]) with 95% confidence intervals (CIs), and Hedges’ g with unequal-variance correction.

Given baseline imbalances in age and MMSE (Table 1) and the well-established age dependence of serum NfL, we performed an age- and sex-adjusted linear regression of log-transformed NfL to mitigate potential confounding by age:

$$\:\mathrm{log}\left({\mathrm{N}\mathrm{f}\mathrm{L}}_{i}\right)=\:{\beta\:}_{0}+\:{\beta\:}_{1}{\mathrm{G}\mathrm{r}\mathrm{o}\mathrm{u}\mathrm{p}}_{i}+\:{\beta\:}_{2}{\mathrm{A}\mathrm{g}\mathrm{e}}_{i}+\:{\beta\:}_{3}{\mathrm{S}\mathrm{e}\mathrm{x}}_{i}+\:{\epsilon\:}_{i}$$

The adjusted group effect is reported as adj. GMR = 𝑒^*β1*^ with 95% CI. As a distribution-free sensitivity we report Mann–Whitney U with Cliff’s δ.

Within-subject perioperative change (overall) was assessed using a paired *t*-test on log(NfL) and reported as the 24 h versus baseline GMR with 95% CIs; a Wilcoxon signed-rank test on the original scale served as a robustness check. Group-differential perioperative change was evaluated using a linear mixed-effects model with a random intercept for subject and a group×time interaction:

$$\:{\mathrm{l}\mathrm{o}\mathrm{g}(\mathrm{N}\mathrm{f}\mathrm{L}}_{it})=\:{{\beta\:}_{0}+\:{\beta\:}_{1}{\mathrm{T}\mathrm{i}\mathrm{m}\mathrm{e}}_{t}+\beta\:}_{2}{\mathrm{G}\mathrm{r}\mathrm{o}\mathrm{u}\mathrm{p}}_{i}+\:{\beta\:}_{3}\left({\mathrm{T}\mathrm{i}\mathrm{m}\mathrm{e}}_{t}\times\:\:{\mathrm{G}\mathrm{r}\mathrm{o}\mathrm{u}\mathrm{p}}_{i}\right)+\:{u}_{i}+{\epsilon\:}_{it}$$

Time was coded as 0 at baseline (before premedication) and 1 at 24 h after surgical start. With Group B as the reference group, exp(β₁) is the post/pre GMR in Group B and exp(β₃) is the ratio of post/pre GMRs (Group A vs. Group B). A covariate-adjusted version additionally included age and sex. Model residuals were inspected visually (Q–Q plots).

To address potential residual confounding by cognition, we performed an exploratory baseline model adding MMSE as a covariate (log[NfL] ~ group + age + sex + MMSE). Given the limited sample size, this MMSE-adjusted analysis was interpreted as a sensitivity check of estimate robustness rather than a definitive covariate-adjusted effect.

The baseline between-group comparison constituted the single primary hypothesis (unadjusted). Secondary families comprised: (a) paired/longitudinal effects and (b) sensitivity/exploratory analyses. Benjamini–Hochberg false discovery rate (FDR; q = 0.05) was applied within each family, and the number of tests per family is reported.

Analyses used complete-case data. Prespecified perioperative exclusions (e.g., MAP < 65 mmHg for > 5 min, blood loss ≥ 500 mL, arrhythmia requiring treatment) were applied uniformly across groups. If > 5% of values were missing in key variables, multiple imputation (mice) was planned as a sensitivity analysis; primary inferences rely on complete cases.

Because sample size was constrained by feasible recruitment, we report the minimum detectable effect size (MDES) for the primary baseline GMR at 80% power and α = 0.05 (Welch approximation on the log scale) to calibrate interpretation of non-significant findings.

## Results

**Fig. 1:**
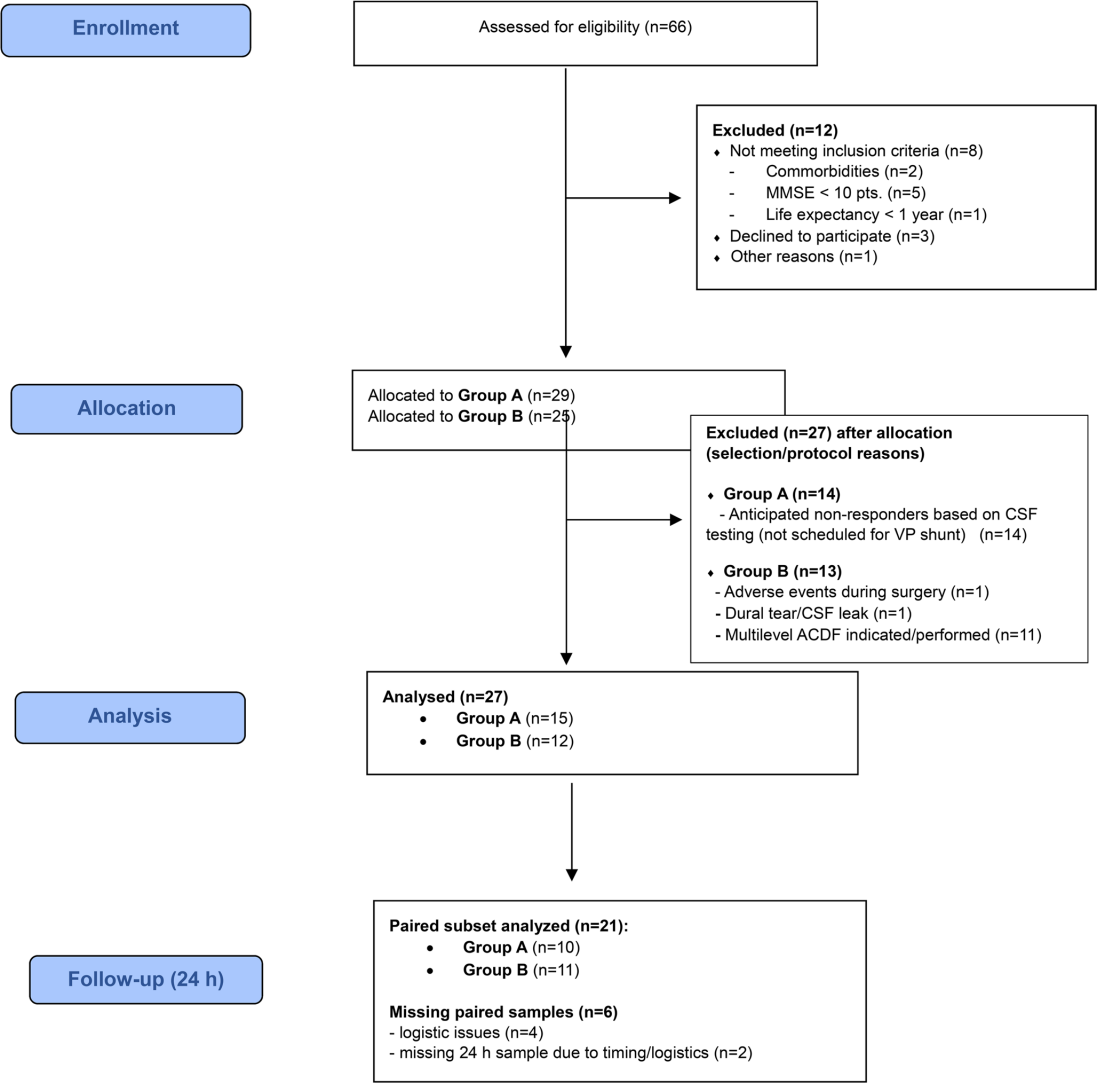
Strengthening the Reporting of Observational studies in Epidemiology (STROBE) flow diagram of participant inclusion, analysis sets, and paired sampling

Between January 2024 and June 2025, 41 adults aged ≥ 50 years were enrolled (Fig. 1). The primary analysis set comprised 27 individuals: 15 anticipated VP-shunt responders with iNPH (Group A) and 12 non-hydrocephalus surgical controls (Group B). A paired subset of 21 participants (10/15 responders; 11/12 controls) contributed baseline (before premedication) and 24-hour post–surgery-start samples. Baseline characteristics are summarized in Table 1. 

**Table 1:** The baseline demographic characteristic and comorbidities

Age (years) – median [IQR]	Group A (*n* = 15)	Group B (*n* = 12)	*P*-value
	73.0 [68.0–75.5]	66.0 [63.3–68.8]	0.008*
Sex (males/females)	8/7	6/6	1.000
Nationality - Czech^1^	15/15 (100%)	12/12 (100%)	1.000
MMSE (pts.) median [IQR]	28 [24.5–29.5]	30 [30–30]	0.002*
Diabetes mellitus	3/15 (20.0%)	2/12 (16.7%)	1.000
Arterial hypertension	7/15 (46.7%)	6/12 (50.0%)	1.000
Ischemic stroke history	1/15 (6.7%)	0/12 (0.0%)	1.000
History of carcinoma	0/15 (0.0%)	0/12 (0.0%)	N/A
Ischemic heart disease, ischemic lower limb disease	2/15 (13.3%)	1/12 (8.3%)	1.000
Other causes of dementia	0/15 (0.0%)	0/12 (0.0%)	N/A

Two-sided tests. Age and MMSE: Mann–Whitney U; categorical variables: Fisher’s exact.

*p < 0.05 indicates between-group difference. ^1^Ethnicity was not separately recorded.

Serum NfL did not differ between anticipated VP-shunt responders and non-hydrocephalus surgical controls on the log scale (Welch’s t, *p* = 0.93), with a geometric mean ratio (responders/controls) of 1.03 (95% CI 0.51–2.10). A non-parametric sensitivity analysis was concordant (Mann–Whitney *p* = 0.965; Cliff’s δ=−0.01). On the original scale, median NfL was 21.05 [10.35–29.76] in responders and 21.80 [13.05–32.93] in controls.

**Fig. 2:**
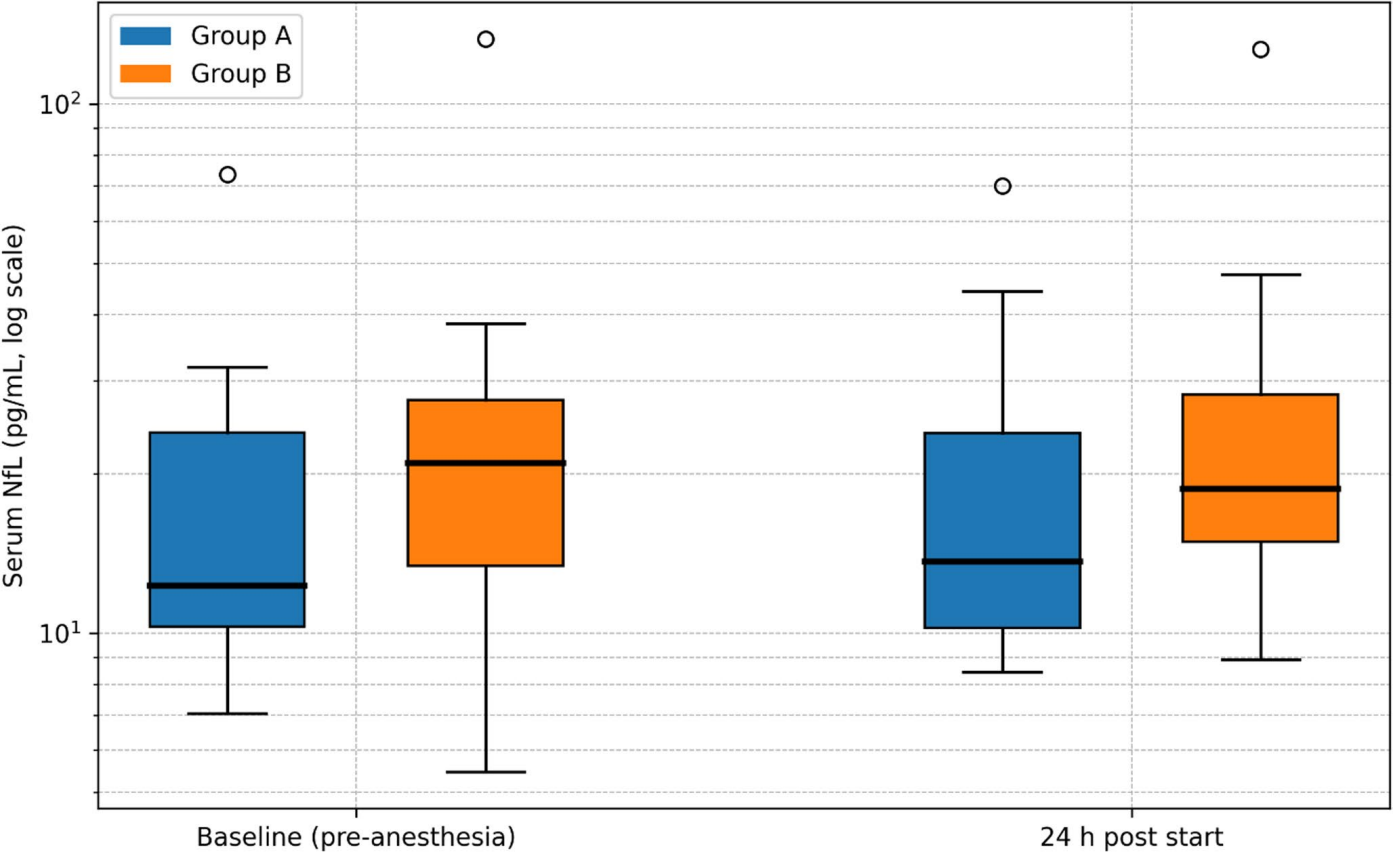
Box plots of serum neurofilament light chain (NfL) at baseline (pre-anesthesia/premedication) and 24 h after surgical start under general anesthesia in VP-shunt responders with iNPH (Group A – blue) and non-hydrocephalus surgical controls (Group B – orange). Boxes indicate the interquartile range (IQR) with the median (horizontal line); whiskers extend to 1.5×IQR; points denote individual observations including outliers. The y-axis is shown on a logarithmic scale. Data are shown for participants with paired samples (*n* = 21)

**Fig. 3:**
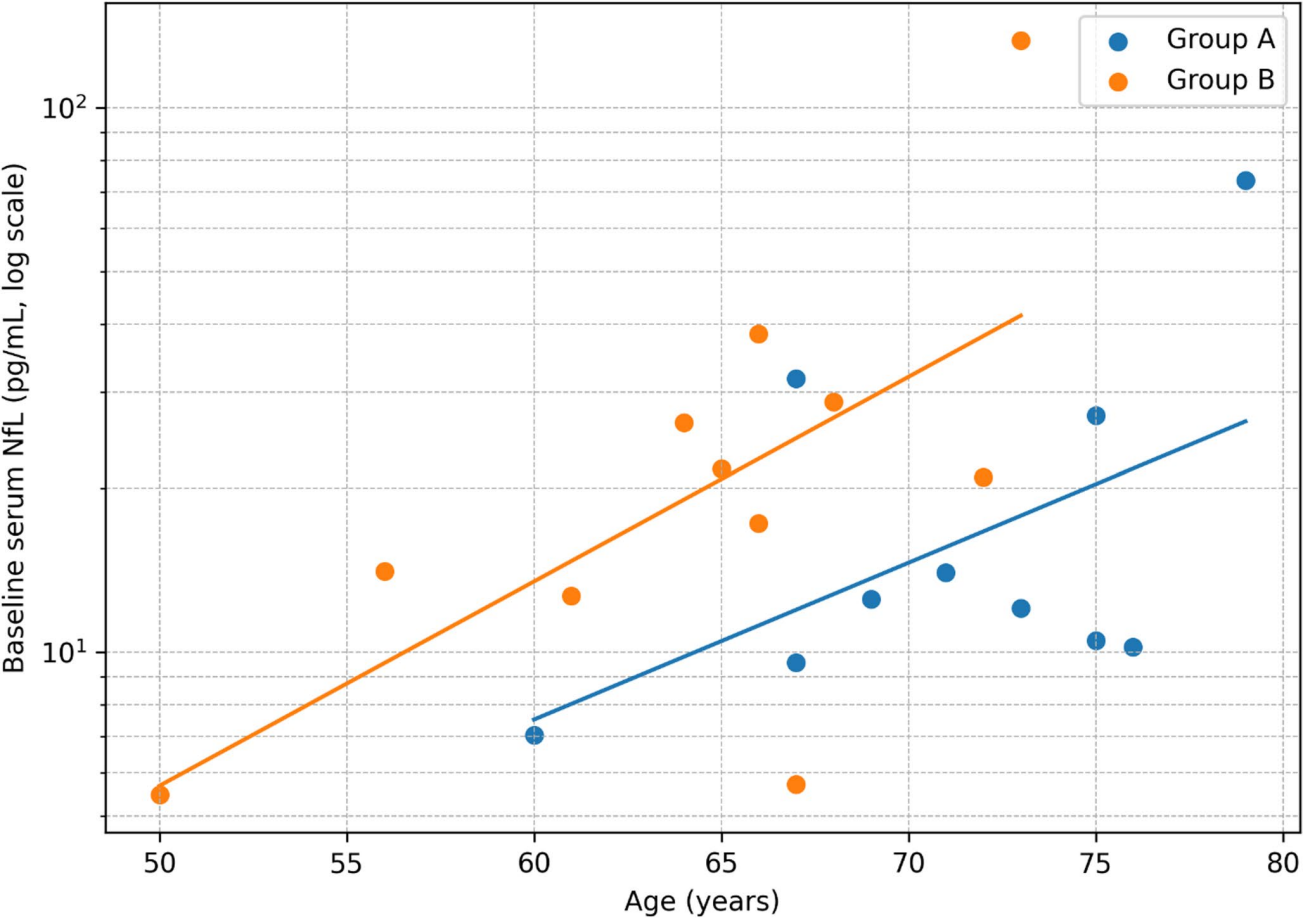
Relationship between age and baseline serum neurofilament light chain (NfL). Scatter plot of individual baseline serum NfL values versus age, displayed on a logarithmic y-axis. Points are stratified by study group (Group A: VP-shunt responders with iNPH - blue; Group B: non-hydrocephalus surgical controls - orange). Lines indicate group-wise fitted trends on the log scale. Data are shown for participants with paired samples (*n* = 21)

Baseline age and MMSE differed between groups (Table 1). In an age/sex-adjusted linear model, the between-group effect remained non-significant with a magnitude comparable to the unadjusted GMR (Fig. 2), suggesting that the null between-group finding was not driven solely by age/sex differences, while acknowledging limited precision and the possibility of residual confounding. The relationship between age and baseline serum NfL on the log scale is shown in Fig. 3.

**Table 2:** Paired comparisons of serum NfL before premedication (NfL_preop) and 24 h after surgical start (NfL_postop) in VP-shunt responders and non-hydrocephalus surgical controls. Δ denotes within-person change on the original scale (pg/mL): Δ = NfL_postop−NfL_preop (median [Q1–Q3] and mean ± SD). GMR is the geometric mean ratio (post/pre) from log-transformed values with 95% CIs. Two-sided p-values from paired t-tests on the log scale

Group	*n*	Δ Median [Q1–Q3]	Δ Mean	Δ SD	GMR	95% CI for GMR	*P*-value
Group A	10	0.32 [-3.06–13.84]	5.64	16.30	1.104	0.78–1.56	0.535
Group B	11	1.46 [-4.01–5.17]	1.51	13.69	1.157	0.77–1.74	0.442
All participants	21	1.40 [-3.51–6.33]	3.48	14.76	1.132	0.89–1.45	0.305

*NfL* = Neurofilament Light Chain; Δ = *NfL*_postop_ – *NfL*_preop_ (reported as median [Q1–Q3] and mean ± SD); *GMR* = geometric mean ratio

**Fig. 4:**
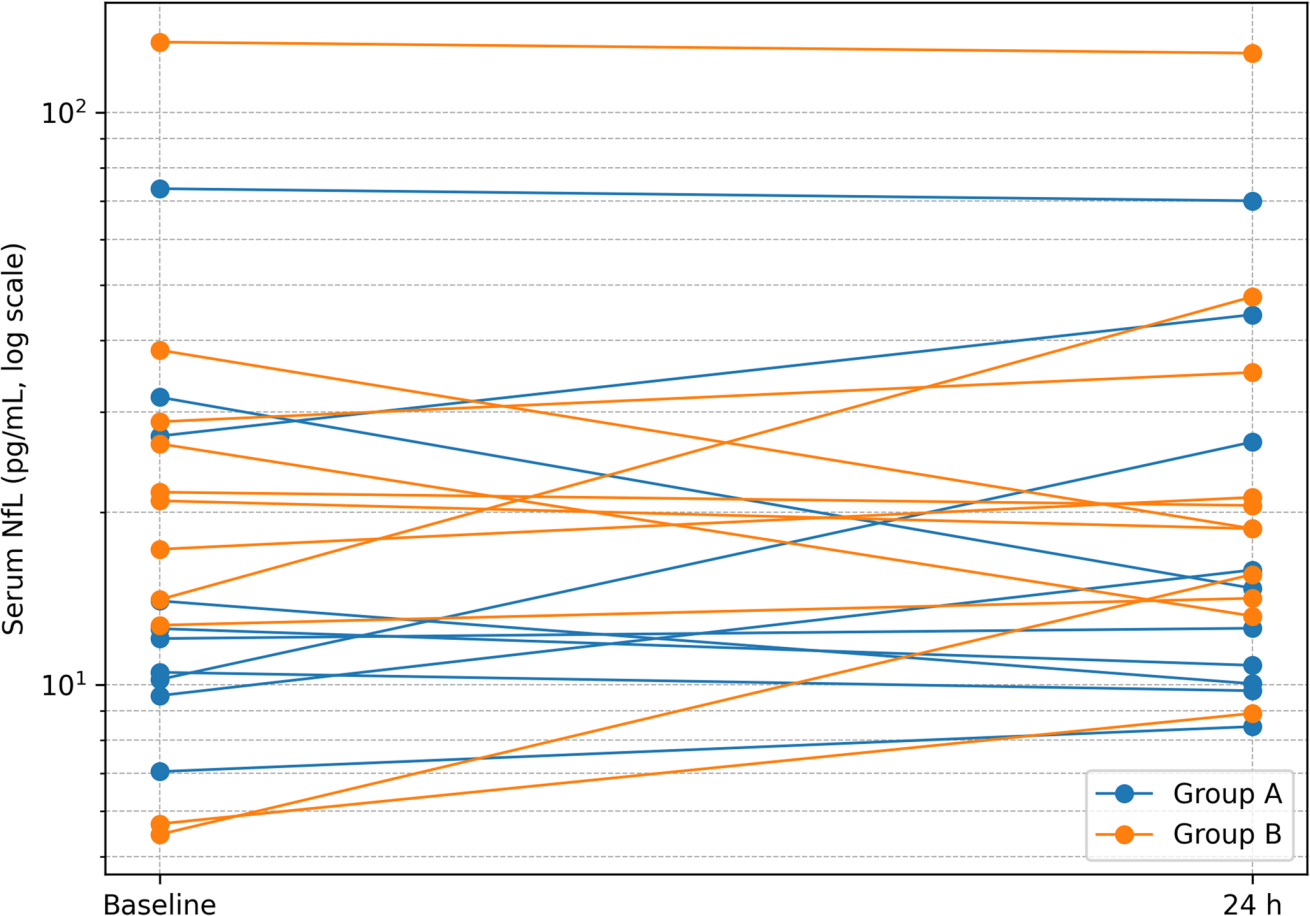
Individual within-subject trajectories of serum neurofilament light chain (NfL) from baseline (before premedication) to 24 h after surgical start under general anesthesia. Each line represents one participant; values are displayed on a logarithmic y-axis. Trajectories are shown separately by group (Group A: VP-shunt responders with iNPH - blue; Group B: non-hydrocephalus surgical controls - orange). Paired samples were available for 10/15 responders and 11/12 controls (total *n* = 21)

Paired sampling was available for 10/15 responders and 11/12 controls (total 21/27), see Table 2. Reasons for missing pairs were logistical/scheduling; there were no assay-related exclusions. Across all participants, serum NfL did not change: paired t-test on the log scale t(20) = 1.05, *p* = 0.305, corresponding to GMR (24 h vs. baseline) = 1.13 (95% CI 0.89–1.45). Sensitivity analyses were concordant (paired t on the original scale *p* = 0.293; Wilcoxon signed-rank *p* = 0.452). Individual trajectories from baseline to 24 h are displayed in Fig. 4.

Despite higher age and lower MMSE in Group A at baseline (Table 1), neither the unadjusted primary contrast nor the age/sex-adjusted model indicated a between-group difference in serum NfL. Similarly, no acute change at 24 h after surgery start under general anesthesia was observed overall or within groups. Findings were consistent across sensitivity analyses, including non-parametric testing and checks for influential observations.

## Discussion

In this prospective cohort of adults ≥ 50 years, serum NfL did not differ between VP-shunt responders with iNPH and non-hydrocephalus surgical controls, and no acute perioperative change was detected at 24 h after surgery start under general anesthesia. The primary between-group contrast was near null (GMR = 1.03; 95% CI 0.51–2.10; *p* = 0.93). In the paired subset, the overall 24 h vs. baseline change was small and non-significant (GMR = 1.13; 95% CI 0.89–1.45; *p* = 0.305). Findings were consistent across covariate adjustment (age/sex) and multiple sensitivity analyses (see Supplementary Methods/Results), supporting that large effects are unlikely within the precision of this dataset. Nevertheless, because Group A was older and had lower MMSE at baseline, we cannot exclude residual confounding or small true differences masked by limited precision, and results should be interpreted as evidence against large effects rather than proof of equivalence. Because serum NfL increases with age—particularly beyond 50 years—we therefore interpreted absolute concentrations in an age-aware manner and focused inference primarily on within-study comparisons and age-adjusted models rather than on universal normative cut-offs.

Across iNPH cohorts, CSF NfL is typically elevated versus surgical controls and associates with worse outcomes after shunting; however, single-marker performance is limited, particularly in blood, where NfL overlaps with levels seen in neurodegenerative and vascular conditions [2, 9–13, 23]. Our null between-group finding in serum is consistent with reports in which plasma/serum NfL did not independently predict postoperative response, whereas multimodal approaches—integrating NfL with Aβ/tau species, structural imaging (e.g., Evans index), and clinical features—achieved better discrimination (e.g., combined model AUC ~ 0.76) [2, 24]. Taken together, current evidence suggests that serum NfL should be interpreted as a contextual injury burden marker rather than a stand-alone diagnostic or exclusionary test in iNPH.

Perioperative biomarker literature further nuances interpretation. Serum NfL commonly increases after surgery—especially in older or high-risk patients—while anesthesia without surgery does not usually produce early rises; reported trajectories often peak at 24–48 h [16–19]. Our sampling at 24 h may therefore precede or straddle the maximal signal, which could partly explain the absent change at 24 h after surgery start under general anesthesia. Moreover, perioperative NfL appears sensitive to procedure-related factors (micro-ischemia, embolic burden, BBB perturbation, systemic inflammation) and to baseline neurodegeneration, both of which vary across populations [20–22]. Clarifying the prognostic meaning of short-term NfL/tau surges—whether they foreshadow delirium severity, covert ischemic injury on MRI, or longer-term cognitive decline—will require harmonized protocols with imaging and neurocognitive follow-up.

Methodological standardization remains a major unmet need. Pre-analytical SOPs (timing relative to induction/extubation, tube type, processing delays, storage), platform cross-validation (Simoa vs. other assays), and agreed-upon age-aware reference frameworks are prerequisites for transportability across centers [22]. In practice, a multimodal panel that combines axonal (NfL), astroglial (GFAP), and neuronal markers (tau/NSE) measured at multiple perioperative timepoints—alongside imaging metrics—may better separate reversible perioperative perturbations from true neuronal injury [18, 19]. From a clinical operations perspective, elevated preoperative NfL could help risk-stratify older patients for enhanced hemodynamic vigilance or embolic mitigation protocols, but such strategies need prospective testing [21].

While results were consistent across sensitivity analyses and age/sex adjustment, residual confounding and small effects cannot be excluded given baseline imbalances and the modest sample size; thus, findings argue against large effects rather than proving equivalence. This study’s strengths include prospective enrollment, an a priori log-scale analytical framework with GMR readouts (appropriate for right-skewed biomarker distributions), and a paired sub-design enabling within-person contrasts. Several limitations temper inference. The modest sample size (primary *n* = 27; paired *n* = 21) yields wide CIs around small effects, and small true differences cannot be excluded (the study was calibrated to detect moderate GMRs). Baseline age and MMSE differed between groups; although age/sex adjustment (and an exploratory MMSE-adjusted model) did not materially change the estimated group effect, the small sample and correlation between age, cognition, and comorbidity burden mean that residual confounding cannot be ruled out. The sampling window included only baseline and 24 h measurements; therefore, delayed peaks (e.g., 24–48 h or later) may have been missed. Paired data were incomplete (10/15 responders; 11/12 controls) for logistical reasons, reducing precision without evidence of assay failure. Platform non-interchangeability may limit external comparability, and the absence of imaging markers and longer-term cognitive follow-up constrains prognostic interpretation. As a design consideration, single-level ACDF was selected as the control procedure to provide comparable exposure to general anesthesia while minimizing direct intracranial or intradural CNS manipulation, thereby improving procedural comparability.

We also address cohort overlap transparently. This study (NCT05399602) partially overlaps with a separately registered cohort (NCT06083233; Cihlo et al., 2025) [25]. Specifically, the 15 VP-shunt responders analyzed here form a subset of the 36 hydrocephalus patients in the prior cohort, whereas the surgical controls were newly recruited. No primary outcomes from the earlier publication are duplicated; the present manuscript addresses distinct aims (between-group serum NfL and short-term change at 24 h after surgery start under general anesthesia). We disclose the magnitude and timing of the overlap (Fig. 1; Supplementary Table S1) to facilitate deduplication in future systematic reviews/meta-analyses. For context only, we cite our prior observation of no serum NfL difference between shunt responders and non-responders; we did not re-analyze or replicate those endpoints here.

Clinically, these findings suggest that serum NfL alone is unlikely to guide shunt decision-making or to serve as an early injury signal in iNPH at 24 h after surgery start under general anesthesia. A pragmatic path forward is to incorporate age-aware serum NfL into multimodal assessment pipelines that already inform iNPH care (clinical phenotype, gait/balance metrics, CSF dynamics, and imaging), reserving CSF biomarkers and advanced imaging for indeterminate cases. For perioperative neuromonitoring, trial designs should test whether targeted intraoperative strategies (hemodynamic optimization, embolic mitigation, anti-inflammatory measures) reduce biomarker release and improve patient-centered outcomes—recognizing that biomarker reductions are only meaningful if accompanied by better recovery trajectories.

In conclusion, within the constraints of sample size and timing, we found no between-group difference in serum NfL between iNPH shunt responders and surgical controls and no early increase at 24 h after surgery start under general anesthesia. Interpretation should remain age-aware and context-dependent. Future work should standardize sampling (including 24–48 h and later timepoints), pre-register adjusted models, integrate multimarker panels with imaging and cognition, and evaluate interventional strategies that target putative mechanisms of perioperative neuronal stress.

## Conclusions

In this prospective cohort of adults aged ≥ 50 years, serum NfL did not differ between VP-shunt responders with idiopathic normal-pressure hydrocephalus (iNPH) and non-hydrocephalus surgical controls, and no acute change at 24 h after surgery start under general anesthesia. Point estimates were near null (primary contrast GMR 1.03; 95% CI 0.51–2.10) and small in the paired subset (24 h vs. baseline GMR 1.13; 95% CI 0.89–1.45), providing no evidence of a clinically meaningful effect within the precision of this study. Taken together with prior work, these findings suggest that serum NfL alone has limited stand-alone diagnostic or prognostic utility for case–control discrimination in iNPH or for detecting very early neuronal injury at 24 h after surgery start under general anesthesia. Interpretation should remain age-aware and integrated with complementary clinical assessments and biomarkers. Larger, multicenter studies with standardized sampling (including later timepoints) and long-term cognitive/imaging follow-up are warranted to define any context-specific role for serum NfL.

## Supplementary Information

Below is the link to the electronic supplementary material.

Supplementary Material 1

## Data Availability

De-identified data and analysis scripts are available upon reasonable request to the corresponding author.
