# Supplementary material for: Serum neurofilament light chain in hydrocephalus and surgical controls: baseline comparison and 24-hour perioperative dynamics under general anesthesia
Source: Neurosurg Rev. 2026 Mar 31;49(1):330. doi: 10.1007/s10143-026-04254-5 (PMC13038456; doi:10.1007/s10143-026-04254-5)
Supplement: Supplementary file 1 — Supplementary Material 1 [file 10143_2026_4254_MOESM1_ESM.docx]

| **Item** | **Current study (NCT05399602)** | **Prior study**  **(Cihlo et al., 2025)** | **Notes** |
| --- | --- | --- | --- |
| **Recruitment window** | 01/2024–12/2024 | 01/2024–09/2024 | Overlap: 01/2024–09/2024 |
| **Total enrolled (overall)** | 41 | 44 | Adults ≥50 y |
| **Included in primary analysis** | 27 | – | Responders=15; Controls=12 |
| **Excluded after enrollment (reasons)** | 14 | – | Detail in flow diagram |
| **Hydrocephalus analyzed (responders only)** | 15 | 36 | All 15 current responders ⊂ prior hydrocephalus |
| **Non-hydrocephalus controls (B)** | 12 | 0 | Controls are new in current study |
| **Paired anesthesia subset** | 10 | N/A | Pre- vs 24 h post anesthesia |
| **Overlap count (overall)** | 22 of 41 (53.7%) | – | Absolute number and % of current enrollment |
| **Overlap – Hydrocephalus** | 15 of 36 (41.7%) | – | =100% of current hydrocephalus |
| **Overlap – Controls** | 0 of 12 (0.0%) | – | No overlap |
| **Non-overlapping new participants** | 19 of 41 (46.3%) | – | 41–22 |
| **Registry cross-reference** | NCT05399602 | NCT06083233 | Cross-linked in text |
| **Primary endpoints (each study)** | Between-group serum NfL; peri-anesthetic change | Shunt responsiveness | Distinct aims |
| **Data reuse/ethics** | Compliant | Compliant | IRB/consent allow secondary analyses |
